# The effect of wine glass size on volume of wine sold: a mega‐analysis of studies in bars and restaurants

**DOI:** 10.1111/add.14998

**Published:** 2020-02-27

**Authors:** Mark Pilling, Natasha Clarke, Rachel Pechey, Gareth J. Hollands, Theresa M. Marteau

**Affiliations:** ^1^ Behaviour and Health Research Unit University of Cambridge Cambridge UK

**Keywords:** Alcohol, choice architecture, glass size, mega‐analysis, multiple treatment reversal design, portion size, purchasing, replication, sales, wine

## Abstract

**Aim:**

To estimate the effects of wine glass size on volume of wine sold in bars and restaurants.

**Design:**

A mega‐analysis combining raw (as opposed to aggregate‐level) data from eight studies conducted in five establishments. A multiple treatment reversal design was used for each data set, with wine glass size changed fortnightly while serving sizes were unaffected, in studies lasting between 14 and 26 weeks.

**Setting and participants:**

Five bars and restaurants in England participated in studies between 2015 and 2018, using wine glasses of five sizes: 250, 300, 370, 450 and 510 ml, with the largest size only used in bars.

**Measurements:**

Daily volume of wine sold by the glass, bottle or carafe for non‐sparkling wine were recorded at bars (594 days) and restaurants (427 days), averaging 4 months per study.

**Findings:**

Mega‐analysis combining data from bars did not find a significant effect of glass size on volume of wine sold compared with 300‐ml glasses: the volume of wine sold using 370‐ml glasses was 0.5% lower [95% confidence interval (CI) = –8.1% to 6.1%], using 450‐ml glasses was 1.0% higher (95% CI = –9.1 to 12.2) and using 510‐ml glasses was 0.4% lower (95% CI = –9.4 to 9.4). For restaurants, compared with 300‐ml glasses, the volume of wine sold using 250‐ml glasses did not show a significant difference: 9.6% lower (95% CI = –19.0 to 0.7). Using 370‐ml glasses the volume of wine sold was 7.3% higher (95% CI = 1.5% to 13.5%); no significant effect was found using 450‐ml glasses: 0.9% higher (95% CI = –5.5 to 7.7).

**Conclusions:**

The volume of wine sold in restaurants in England may be greater when 370‐ml glasses are used compared with 300‐ml wine glasses, but may not be in bars. This might be related to restaurants compared with bars selling more wine in bottles and carafes, which require free‐pouring.

## Introduction

The adverse health effects of alcohol consumption are well described. It is the fifth largest contributor to premature death in high‐income countries and seventh world‐wide 1. Reducing the size of glasses, serving sizes and containers in which alcohol is sold comprise an underexplored set of interventions for reducing consumption. We focus here on wine glass size to assess whether serving wine in smaller glasses, without altering portion sizes, reduces the volume of alcohol sold.

Wine glasses have increased in size almost sevenfold during the last 300 years, with the most marked increase a doubling in size since 1990 2. During this time, the amount of wine consumed in England quadrupled, although the number of wine consumers remained constant 3, an association compatible with the hypothesis that larger wine glasses increase the amount of wine consumed. Such a hypothesis is also in keeping with the large body of evidence for the portion size effect in relation to food: the larger the portion, package or tableware, the more individuals consume 4.

The first study to assess the impact of wine glass size on sales found that serving wine (typically a 175‐ml measure) in 370‐ml compared with 300‐ml glasses increased sales by 9.4% 5. In a series of further studies conducted in bars and restaurants, a mixed pattern of effects was observed 6, 7. Where significant effects have been observed these consistently showed larger wine glasses increased sales 6, 7 but uncertainty remains about the contexts in which these effects might occur. An analysis combining data across these studies could clarify the effects on sales of altering wine glass size in restaurants and bars.

Wine sales in bars and restaurants are either of fixed serving sizes when sold by the glass, or for free‐pouring by customers or by staff when sold by the bottle or carafe. Sales by the bottle or carafe are more common in restaurants than bars. The size of wine glasses might affect sales differently, depending on whether it is sold by the glass or by the bottle or carafe.

Regarding sales by the glass, any effect of glass size on consumption may be driven by perceptual effects. People tend to consume in units; for example, having one cup of coffee, one slice of cake or one glass of wine—known as the unit bias heuristic 8. The same portion size in a larger glass appears smaller than when presented in a smaller glass 9. If a fixed serving size of wine in a larger glass is perceived as less than a glass, this might increase the likelihood of people buying additional servings, thereby increasing sales of wine by the glass when it is served using larger glasses. An indirect test of this hypothesis in a laboratory‐based study failed to find evidence to support it 10.

By contrast, when wine is sold by the bottle or carafe, this involves free‐pouring. i.e. not being decanted into glasses in a fixed serving size using a thimble measure or a standardized mark on the glass 5, 6, 7. Larger wine glasses afford or allow greater portions to be poured from bottles or carafes. Furthermore, when asked to pour a standard serving size, people tend to over‐pour, an effect that increases with the size of the glass and the bottle 11. If these larger portions are still perceived to be ‘a glass’, according to the unit bias heuristic, greater purchasing and consumption would be expected with larger glasses. Consistent with these observations, more wine is predicted to be poured, consumed and sold when it involves free‐pouring.

The aim of the current study was to provide the most robust estimate to date of the effect size of wine glass size on sales—a proxy for consumption—by conducting a mega‐analysis on previously published data sets. Given the potential different mechanisms for effects of glass size on wine sold by the glass versus by the bottle or carafe—the latter more common in restaurants than in bars—we sought to estimate the effects separately for bars and restaurants.

## Methods

### Study population

We report here a mega‐analysis (defined as combining the raw, as opposed to aggregated‐level, data from multiple studies 12) combining eight data sets from studies conducted between 2015 and 2018 in five independent eating and drinking establishments in Cambridge, England. This represents all available studies, all produced by our research group.

To identify further studies eligible for inclusion in the analysis, an electronic search was conducted. The search was carried out in PsycINFO, MEDLINE and Google Scholar (14 October 2019) and included free‐text terms such as ‘wine’, ‘glass’, ‘size’ and ‘sales’ (see Supporting information for search strategy). The search identified 110 records for title and abstract screening. To meet eligibility criteria, studies were required to assess the impact of different wine glass sizes on sales or consumption. This search retrieved all the studies included in the manuscript. Forward and backward citation searches were also conducted on the included papers and two ineligible but relevant studies on wine glass size 2, 10. No further studies were identified as eligible for inclusion in the analysis.

This pre‐registered (https://osf.io/uarnz/) analysis combines all previously published 5, 6, 7 data. Full details of the procedures used in collecting these eight data sets are available in three publications 5, 6, 7, summarized in Table 1. Daily wine sales were recorded in studies lasting between 14 and 26 weeks (representing 594 days from bars and 427 days from restaurants), with wine glasses changed in size over fortnightly periods. Within each study the style of glassware was kept constant, although there were some minor differences between studies. Capacities (the total volume of liquid when filled to the rim) of wine glasses used in the studies were 250, 290, 300, 350, 370, 450 and 510 ml. Data were combined from two pairs of glasses that differed by less than 10%: 290 and 300 ml, and 350 and 370 ml, resulting in five different wine glass capacities for the analysis. For simplicity, 300 ml is used to describe 290‐ and 300‐ml glasses, and 370 ml is used to describe 350‐ and 370‐ml glasses.

**Table 1:** Establishment and data set characteristics

Data set	Establishment	Setting	Study date	Glass sizes (ml)^a^	Sales by‐the‐glass (%)	Days
1	I	Bar	2015	250(C) 300(A) 370 (B)	93	112
2	III	Bar	2016	300(C) 510(B)	88	98
3	II	Bar	2016	300(C) 370(A) 510 (B)	88	123
4	II	Bar	2018	290(C) 350(A) 450 (B)	90	125
5	IV	Bar	2018	290(C) 350(A) 450 (B)	91	136
6	I	Restaurant	2015	250(C) 300(A) 370 (B)	63	112
7	V	Restaurant	2017	290(C) 350(A) 450 (B)	66	126
8	V	Restaurant	2018	290(C) 350(A) 450 (B)	67	189

a See Supporting information, Table S1 for details on each design, using this ABC key.

In keeping with UK legal requirements 13 all establishments served wine either by the bottle (750 ml) or by the glass, in two or three volumes from three specified quantities (125, 175, 250 ml). At establishments I and V, wine was also available by the carafe (500 or 1000 ml). Sparkling wines sales were excluded from analysis, as these were served in a fixed type of glass, meaning that the glass size could not be modified.

### Measures

Effect sizes (percentage change) were based on the daily sales of non‐sparkling wine (ml) from previously reported studies. At establishment I there were separate bar and restaurant areas. The other establishments comprised either a bar or a restaurant. Establishment II (bar) provided data sets in both 2016 and 2018 (data sets 3 and 4, Table 1). Establishment V (restaurant) provided data sets in both 2017 and 2018 (data sets 7 and 8). Establishments I and V offered wine by the glass in 125‐ and 175‐ml measures, while the other establishments additionally offered a 250‐ml measure. At each establishment, glass size was changed over fortnightly periods using a multiple treatment reversal design (for further details, see Table 1 and Supporting information, Table S1). The primary outcome was the daily volume of wine (ml) sold from till data.

### Analysis

The analysis plan was pre‐registered https://osf.io/uarnz/.

The analysis was planned to estimate volume daily sales of wine across (i) bars only and (ii) restaurants only using all eight data sets. Glass sizes of 300 ml (which, as noted above, also included glass sizes of 290 ml) were used as the reference level, as this was the only size used at every site. Analyses included five different wine glass capacities: 250, 300 (comprising 290 and 300), 370 (comprising 350 and 370) and 450 and 510, with the latter size only used in bars. A secondary analysis examined volume sales of wine for bars and restaurants combined.

Separate regressions for each of the analyses were used to predict the natural log of the daily wine sales volume (in ml) from glass size. These analyses included main factors for year, with random effects by establishment. For the additional analysis across both bars and restaurants, establishment type (i.e. bar or restaurant) was used as a fixed factor. Analyses also included covariates used in the analyses of the original studies, such as the busyness of the establishment, as measured by the log of their daily sales of products excluding wine. Dummy variables indicating day of the week and month adjusted for weekly and seasonal time trends. Weather variables (e.g. daily temperature at 5 p.m., daily rainfall, daily minutes of sunshine) were examined as candidate covariates in each analysis, with temperature included in the final models. Other contextual variables such as public and school holidays were also examined and included. Finally, given the impact of major sporting events on alcohol sales [e.g. the 2016 Union of European Football Associations (UEFA) European Championship, the 2018 World Cup], variables for these were examined and included in the analysis for each establishment—these potential covariates have proved useful in some of the previously reported studies 5, 6, 7. Data from periods during which glass changeover could not be confirmed were excluded from the analysis. Analysis of each data set needed to control for heteroscedasticity by using the Generalized Additive Model for Location, Scale and Shape (GAMLSS) models 14, and therefore the different analyses sometimes required different explanatory mean terms or variance terms. Alternative model formulations were compared [Akaike's information criterion (AIC)] and used to confirm the robustness of results. Model diagnostics were checked (i.e. residuals, QQplots, worm plots, pacf) and found to be good, with no outliers apparent.

### Public involvement in research

This research relied on previous studies which gained the cooperation of commercial establishments in the public domain to discuss acceptable study designs and agree to the release of commercially sensitive information.

## Results

**Figure 1:**
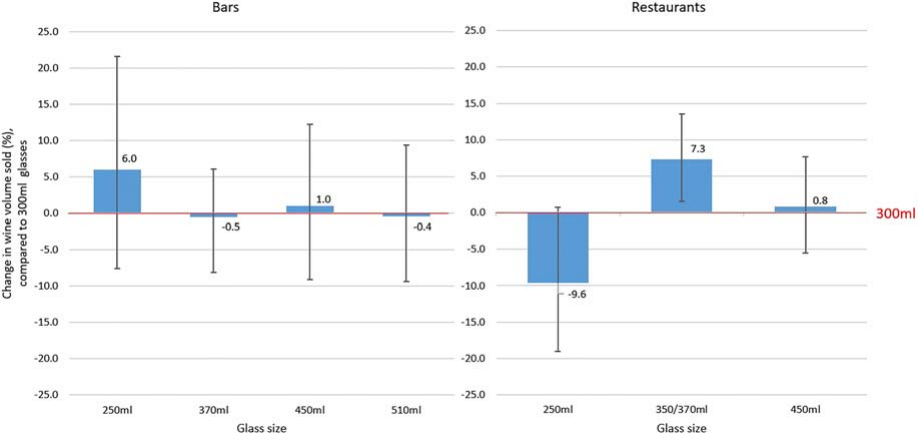
Predicted percentage change (with 95% confidence interval) in daily wine volume sales from 300‐ml glasses in (i) bars and (ii) restaurants. The red line indicates zero change compared to sales with 300‐ml glasses. [Colour figure can be viewed at wileyonlinelibrary.com]

Table 2 summarizes the percentage change in daily wine sales between wine glass sizes in each of eight data sets by type of establishment, i.e. bar or restaurant. Figure 1 summarizes the mean and 95% confidence intervals (CI) for wine sales from each glass size relative to 300 ml glasses, by establishment.

**Table 2:** Regression models assessing the impact of wine glass size on log volume of daily wine sold for (i) bars‐only, (ii) restaurants‐only and (iii) overall.^a^

	Bar estimate [*P*‐value]	%change (95% CI)	Restaurant estimate [*P*‐value]	%change (95% CI)	Overall estimate [*P*‐value]	%change (95% CI)
Modelling of the mean log wine volume						
(intercept)	2.876 [< 0.001]***	NA	4.814 [< 0.001]***	NA	4.054 [< 0.001]***	NA
Glass size 250 ml	0.058 [0.408]	6.0% (−7.6 to 21.6)	−0.101 [0.068]†	−9.6% (−19.0 to 0.7)	−0.040 [0.326]	−4.1% (−11.9 to 4.3)
300 ml	Ref		Ref		Ref	
370 ml	−0.013 [0.728]	−0.5% (−8.1 to 6.1)	0.071 [0.013]*	7.3% (1.5 to 13.5)	0.042 [0.062]†	4.3% (−0.2to 9.1)
450 ml	0.010 [0.850]	1.0% (−9.1 to 12.2)	0.009 [0.784]	0.9% (−5.5 to 7.7)	0.005 [0.867]	5.0% (−5.2 to 6.5)
510 ml	−0.004 [0.926]	−0.4% (−9.4 to 9.4)	NA	NA	0.028 [0.537]	2.8% (−5.9 to 12.4)
Busyness level (log)	0.923 [p < 0.001]***	151.7% (126.4 to 180.0)	0.685 [< 0.001]***	100.3% (76.9 to 126.9)	0.711 [< 0.001]***	103.5% (89.7 to 118.3)
Day: Monday	Ref		Ref		Ref	
Tuesday	0.018 [0.779]	1.8% (−10.2 to 15.5)	0.023 [0.671]	2.3% (−7.9 to 13.7)	0.050 [0.242]	5.1% (−3.3 to 14.3)
Wednesday	0.071 [0.246]	7.3% (4.8 to 21.0)	0.205 [< 0.001]***	22.8% (9.5 to 37.6)	0.179 [< 0.001]***	19.6% (10.1 to 30.0)
Thursday	0.115 [0.071]*	12.2% (9.5 to 27.2)	0.150 [0.011]*	16.2% (3.5 to 30.4)	0.180 [< 0.001]***	19.7% (10.0 to 30.1)
Friday	0.145 [0.044]*	15.6% (0.4 to 33.1)	0.411 [< 0.001]***	50.7% (35.4 to 67.8)	0.380 [< 0.001]***	46.2% (34.5 to 58.9)
Saturday	0.149 [0.079]†	16.1% (1.7 to 37.1)	0.455 [< 0.001]***	57.6% (36.5 to 82.0)	0.438 [< 0.001]***	54.9% (40.0 to 71.5)
Sunday	−0.054 [0.353]	−5.3% (15.5 to 6.2)	−0.129 [0.042]*	−12.1 (−22.4 to −0.5)	−0.047 [0.276]	−4.6% (−12.4 to 3.8)
Year: 2015	Ref		NA		Ref	
2016	0.329 [< 0.001]***	39.0% (25.0 to 54.5)	Ref		0.313 [< 0.001]***	36.7% (25.6 to 48.8)
2017	NA		0.089 [0.041]*	9.3% (0.4 to 19.0)	0.135 [< 0.001]***	14.5% (6.6 to 23.0)
2018	0.136 [0.007]**	14.6% (3.8 to 26.6)	0.028 [0.480]	2.9% (−4.9 to 11.2)	0.089 [0.004]**	9.3% (3.0 to 16.0)
Temperature (C)	−0.016 [< 0.001]***	−1.5% (−2.2 to −1.0)	−0.007 [0.010]*	0.7% (−1.2 to −0.2)	−0.009 [< 0.001]***	−0.9% (−1.3 to −1.0)
Bank holiday, no	Ref		Ref		Ref	
Yes	−0.069 [0.429]	−6.6% (−21.2 to 10.7)	0.053 [0.526]	5.4% (−10.4 to 24.0)	0.016 [0.741]	1.6% (−7.5 to 11.6)
School holiday, no	Ref		Ref		Ref	
Yes	−0.007 [0.840]	−0.7% (−7.2 to 6.2)	−0.058 [0.039]*	−5.6% (−10.6 to −0.3)	−0.021 [0.369]	−2.0% (−6.3 to 2.5)
World Cup or UEFA football						
No	Ref		Ref		Ref	
Yes	−0.120 [0.005]**	−11.3% (−18.3 to −3.7)	−0.075 [0.194]	−7.3% (−17.2 to 3.9)	−0.088 [0.009]**	−8.5% (−14.3 to −2.2)
England football, no	Ref		Ref		Ref	
Yes	−0.241 [0.035]*	−21.4% (−37.2 to −1.7)	0.119 [0.248]	12.6% (−7.9 to 37.8)	−0.076 [0.409]	−7.3% (−22.6 to 11.0)
Setting, bars	NA		NA		Ref	
Restaurants	NA		NA		0.676 [< 0.001]***	96.6% (87.5 to 106.2)

† *P*‐value < 0.1;

* *P*‐value < 0.05;

** *P*‐value < 0.01;

*** *P*‐value < 0.001.

a The outcome is the daily volume of wine sold on the natural log scale. Parameter 95% confidence intervals (CI) and *P*‐values, respectively, appear in parenthesis and in square brackets. Ref = reference category; NA = not applicable; UEFA = Union of European Football Associations.

There were no significant differences in wine sales by glass size in bars, with three of the four contrasts tested suggesting absolute changes of 1% or less, with wide CIs.

Wine sales in restaurants were higher when glass size was increased from 300 ml (reference) to 370 ml (7.3%, 95% CI = 1.5 to 13.5, *P* = 0.013). Comparing sales with 250‐ml glasses to sales with 300‐ml glasses suggested lower sales with the former glasses, albeit with a CI that crossed zero (−9.6%, 95% CI = –19.0 to 0.7, *P* = 0.068). While the pattern of results for restaurants seems relatively linear between glass sizes of 250 to 370 ml, this pattern did not hold for the largest glass size tested in this context (450 ml), for which there was no significant difference in sales compared to using 300‐ml glasses (0.9%, 95% CI = –5.5 to 7.7, *P* = 0.784).

Similar patterns of results by covariates were observed to those reported in previous studies; namely, lower sales of wine with increasing temperature and sales on Friday/Saturday being much higher than on Mondays. There were also different variances on different days of the week, as seen in the separate studies. Football events negatively affected wine sales in bars, but not in restaurants.

### Secondary analysis

There were no statistically significant differences in wine sales by glass size when combining data from bars and restaurants. Wine sales in restaurants were almost double those in bars (96.6% higher, 95% CI = 87.5 to 106.2, *P* < 0.001). Variability of sales in bars was also significantly greater than in restaurants (Supporting information, Table S3 **,** *P* < 0.001).

## Discussion

The results of this mega‐analysis showed no significant effect on daily wine sales of wine glass size in bars. For restaurants, using 370‐ml glasses increased sales by 7.3% over 300‐ml glasses (95% CI = 1.5% to 13.5%). The results also suggested that using 250‐ml glasses might decrease sales compared to 300‐ml glasses, although confidence intervals crossed zero (−9.6%, 95% CI = –19.0 to 0.7). The results showed no significant effect on wine sales comparing 450‐ with 300‐ml glasses (0.9%, 95% CI = –5.5 to 7.7). The effect observed in restaurants for 370‐ over 300‐ml glasses—if sustained—has the potential to make a meaningful contribution to reducing alcohol consumption in licensed premises. The uncertainty around the estimated effect sizes is, however, considerable, ranging from 1.5 to 13%.

There are several possible explanations for the main findings. The absence of an overall significant effect in bars may reflect the lower power of the study in its ability to detect an effect of wine glass size when sold by the glass. Two factors could have contributed to this. First, there was greater variation in wine sales in bars compared to restaurants which would contribute to a much smaller effect size. This may be due to external factors, such as football events, causing greater variation in wine sales in bars than restaurants, for which modelling can only partially mitigate.

Secondly, any effect might be smaller in bars than in restaurants if the main mechanism by which glass size affects consumption is through free‐pouring of wine from bottles or carafes, which is more common in restaurants than bars. Support for this explanation is provided by a laboratory study 10 that found limited differences in micro‐drinking behaviours and perceptions between drinking 175 ml of wine from 370‐ and 250‐ml glasses.

An alternative explanation for no significant effect of wine glass size on wine sales being observed in bars is that it reflects a true null effect: wine glass size has no impact on wine sales in bars.

Regarding the effect of wine glass size on sales in restaurants, this was evident in one of three glass size comparisons: between 300 and 370 ml and suggestive in another: between 300 and 250 ml, but not evident in the third: 370 and 450 ml. First, to explain why effects were evident in some comparisons in restaurants but not in bars: this may reflect greater sales from bottles and carafes in restaurants (35%) compared with bars (8%), thereby affording more opportunity for free‐pouring by customers and waiting staff. In line with this, several laboratory‐based studies observe that more wine is poured from bottles when larger glasses are provided 15, 16. While similar effects were observed in another study of bar staff pouring shots and mixed drinks, these effects were not observed for wine 17.

Secondly, to explain why the effects are not linear, this may reflect different responses to common and unusual sizes. Glass sizes of 300 and 350 ml were commonly used in the study establishments before their participation. For these glass sizes, differences may not have been apparent to drinkers avoiding any conscious response. In contrast, 450‐ and 510‐ml glasses may have been perceived as noticeably larger, prompting conscious counter behaviour to reduce excess consumption such as drinking more slowly or pouring with greater caution. This could reflect a unit bias heuristic, whereby portions in glasses sized between 250 and 370 ml are perceived as ‘a typical glass of wine’, even if more wine is poured into the glasses as they increase in size. As such, within this range, increasing glass size might increase purchases and consumption. If, however, larger glass sizes—450 and 510 ml—are regarded as holding more than a typical glass of wine, then after this size threshold, individuals may adapt their behaviour, resulting in a non‐linear relationship between glass size and purchasing.

### Strengths and limitations

The strength of this study is that it provides the most robust estimate to date of the impact of wine glass size upon sales, a proxy for consumption, by combining all known existing data sets that systematically compare wine sales when wine is served in glasses of varying sizes. In addition, the plan of analysis was pre‐registered and the observations included in the analyses were derived from pre‐registered studies, all of which are published in peer‐reviewed journals.

There are several limitations. First, the study is based on a relatively small number of observations which contribute to the uncertainty around the estimated effect of wine glass size on consumption, inferred from sales. Secondly, the data sets are generated as part of studies with non‐randomized designs which might increase the risk of some other types of biases. For example, as blinding was not possible higher sales of wine when larger glasses were used may reflect staff behaviour, such as promoting wine sales in bottles rather than by the glass when larger glasses were used. We have no reason to suspect this was the case, with staff requested to behave as usual, but the studies, as designed, cannot exclude this possibility. Thirdly, the outcome measure, wine sales, is a proxy for actual consumption, albeit a probably strong predictor of actual consumption in licensed premises. For example, in a recent study 18 less than 1% of wine purchased in a bar setting was left undrunk (personal communication: e‐mail from I. Kersbergen PhD in November 2019). Fourthly, data on sales of other alcoholic drinks were not consistently available, so we were unable to determine whether the changes to wine glass size might also be associated with changes to purchasing of other alcoholic drinks. This is an important consideration to address in further studies. Fifthly, the establishments in which the data sets were collected varied in ways known—e.g. glassware design—and unknown, that could have affected the results.

### Implications for research and policy

Further research on the impact of wine glass size on consumption in bars and restaurants is warranted. This would include extending current evidence to include bars and restaurants in parts of England that are more deprived than Cambridge as well as in other countries, and from other research groups. Research on the impact of wine glass size on consumption is also required in other settings, particularly in homes where most alcohol, including wine, is consumed. Such studies might include the impact on consumption of using smaller wine glasses in conjunction with bottles or boxes of different sizes including those smaller than 750 ml as well as larger.

Regulating wine glass capacity merits consideration for inclusion in local licensing regulations for reducing drinking outside the home. In addition to contributing to population‐level interventions to reduce consumption 19, capping wine glass sizes in licensed premises has the potential to change the social norm for the size of glass from which wine is consumed 20.

## Conclusion

The volume of wine sold in restaurants in England may be greater when 370‐ml rather than 300‐ml wine glasses are used, but may not be in bars. This might reflect more wine sales in restaurants in bottles and carafes, requiring free‐pouring. This effect may also be present, but to a lesser extent, when comparing 300‐ml wine glasses with 250‐ml glasses, but it may not be evident with wine glasses larger than 370 ml.

## Declaration of interests

None.

## Supporting information


**Table S1** Establishment names from previous publications.
**Table S2** Number of observations (days), by glass size, per study.
**Table S3** Regression model assessing the impact of wine glass size on log volume of wine sold for i) bars, ii) restaurants and iii) overall^a^ – components modelling the variance.
