# Supplementary material for: The effect of wine glass size on volume of wine sold: a mega‐analysis of studies in bars and restaurants
Source: Addiction. 2020 Feb 27;115(9):1660–7. doi: 10.1111/add.14998 (PMC7496108; doi:10.1111/add.14998)
Supplement: Supplementary file 1 — Table S1 Establishment names from previous publications. Table S2 Number of observations (days), by glass size, per study. Table S3 Regression model assessing the impact of wine glass size on log volume of wine sold for i) bars, ii) restaurants and iii) overalla – components modelling the variance. [file ADD-115-1660-s001.docx]

Table S1: Establishment names from previous publications

| Dataset | Paper | Establishment name in published papers | Free-pouring sizes (cl) † | Design (fortnights)†† |
| --- | --- | --- | --- | --- |
| 1 | Pechey et. al. (2016) | Bar | 75 | ABAC**A***BACA |
| 2 | Pechey et. al. (2017) | Bar 2 | 75 | CBCBCBC |
| 3 | Pechey et. al. (2017) | Bar 1 | 75 | ABACABACA |
| 4 | Clarke et. al. (2019) | Study 4 | 75 | ABACABACA |
| 5 | Clarke et. al. (2019) | Study 3 | 75 | ABACABACA |
| 6 | Pechey et. al (2016) | Restaurant | 75 | ABAC**A***BACA |
| 7 | Clarke et. al. (2019) | Study 1 | 50/75/100 | ABACABACA |
| 8 | Clarke et. al. (2019) | Study 2 | 50/75/100 | ABACABACABACA |

†Size of the bottle (75) or carafe (50,100).

††Table 1 gives the glass sizes in each establishment for these codes.

*These fortnights (bold) were omitted due to protocol violation.

Table S2: Number of observations (days), by glass size, per study

| Dataset | All | 250ml | 290/300ml | 350/370ml | 450ml | 510ml |
| --- | --- | --- | --- | --- | --- | --- |
| 1 | 112 | 29 | 56 | 27 |  |  |
| 2 | 98 |  | 56 |  |  | 42 |
| 3 | 123 |  | 28 | 67 |  | 28 |
| 4 | 125 |  | 26 | 71 | 28 |  |
| 5 | 136 |  | 26 | 81 | 29 |  |
| 6 | 112 | 29 | 56 | 27 |  |  |
| 7 | 126 |  | 70 | 28 | 28 |  |
| 8 | 189 |  | 98 | 49 | 49 |  |

Table S3: Regression models assessing the impact of wine glass size on log volume of wine sold for i) bars, ii) restaurants and iii) overall^a^ – components modelling the variance

|  | Bar  Estimate  [p-value] | Restaurant  Estimate  [p-value] | Overall  Estimate  [p-value] |
| --- | --- | --- | --- |
| Modelling of the variance wine volume (log link) |  |  |  |
| (intercept) | -0.801 [<0.001]*** | -1.128 [<0.001]*** | -0.789 [<0.001]*** |
| Glass size 250ml | -0.226 [0.123] | -0.113 [0.435] | -0.170 [0.096]. |
| 300ml | Ref | Ref | Ref |
| 370ml | -0.153 [0.031]* | -0.223 [0.016]* | -0.184 [<0.001]*** |
| 450ml | -0.261 [0.021]* | -0.152 [0.090]. | -0.165 [0.024]** |
| 510ml | -0.286 [0.006]** | NA | -0.307 [0.002]** |
| Day Monday | Ref | Ref | Ref |
| Tuesday | 0.056 [0.609] | 0.053 [0.689] | 0.033 [0.705] |
| Wednesday | -0.077 [0.483] | 0.165 [0.224] | -0.015 [0.861] |
| Thursday | -0.064 [0.562] | 0.193 [0.144] | -0.019 [0.828] |
| Friday | -0.396 [<0.001]*** | -0.309 [0.022]* | -0.366 [<0.001]*** |
| Saturday | -0.437 [<0.001]*** | -0.573 [<0.001]*** | -0.509 [<0.001]*** |
| Sunday | -0.231 [0.040]* | -0.217 [0.121] | -0.157 [0.071]. |
| School Holiday No | NA | Ref | NA |
| Yes | NA | -0.152 [0.087]. | NA |
| England Football No | Ref | NA | Ref |
| Yes | 0.402 [0.024]* | NA | 0.419 [0.006]** |
| Setting Bars | NA | NA | Ref |
| Restaurants | NA | NA | -0.294 [<0.001]*** |

Significance key: ‘.’ For p-value<0.1,’*’ for p-value <0.05, ‘**’ for p-value<0.01. ‘***’ for p-value<0.001. ^a^The outcome is the daily volume of wine sold on natural log scale. Parameter 95% confidence intervals and p-values respectively appear in parenthesis and in square bracket. “NA” is not applicable.

**S4. Search strategy**

**All searches combined = 117**

**Removing duplicates = 110**

**MEDLINE SEARCH - OVID MEDLINE (R) and In process& other non-indexed citations 1946 to present**

**Ran on 14.10.19**

**Records returned = 20**

S1. wine* OR champagne*

S2. ((glass* OR drinkware) adj9 (size* OR capacit* OR volume*))

S3. 1 AND 2

S4. (drink* OR drunk* OR consum* OR sale* or sold OR purchas*)

S5. 3 AND 4

**PsycINFO (EBSCOhost) 1806 to 2019**

**Ran on 14.10.19**

**Records returned = 18**

S5. 3 AND 4

S4. (drink* OR drunk* OR consum* OR sale* or sold OR purchas*)

S3. 1 AND 2

S2. ((glass* OR drinkware) AND (size* OR capacit* OR volume*))

S1. wine* OR champagne*

**Google Scholar**

**Ran on 14.10.19**

**Records returned = 79**

(wine OR champagne) AND ((glass OR drinkware) NEAR/9 (size OR capacity OR volume)) AND (drink OR drunk OR consume OR sale or sold OR purchase)
